# Gabapentin for chronic refractory cough: A system review and meta-analysis

**DOI:** 10.1016/j.heliyon.2023.e15579

**Published:** 2023-04-18

**Authors:** Sheng Xie, Meiling Xie, Yongchun Shen, Deyun Cheng

**Affiliations:** a Department of Pulmonary and Critical Care Medicine, Chengdu First People's Hospital, Sichuan, China; b Department of Traditional Chinese Medicine, Sichuan Electric Power Hospital, Sichuan, China; c Department of Pulmonary and Critical Care Medicine, West China Hospital, Sichuan University, Sichuan, China

**Keywords:** Chronic refractory cough, Gabapentin, Meta-analysis, Efficacy, Safety

## Abstract

**Objective:**

To evaluate the efficacy and safety of gabapentin in the treatment of chronic refractory cough by Meta-Analysis.

**Methods:**

Literatures were retrieved from PubMed, Embase (OvidIP), Cochrane Library, CNKI, VIP, Wanfang Database and China Biomedical Management System and eligible prospective studies were screened. Data were extracted and analyzed by using RevMan 5.4.1 software.

**Results:**

Six articles (2 RCTs and 4 prospective studies) with 536 participants were finally included. Meta-analysis showed that gabapentin was better than placebo in cough-specific quality of life (LCQ score, MD = 4.02, 95%CI [3.26,4,78], Z = 10.34, P < 0.00001), cough severity (VAS score, MD = −29.36, 95% CI (-39.46, -19.26), Z = 5.7, P < 0.00001), cough frequency (MD = -29.87, 95% CI [- 43.84, -15.91], Z = 4.19, P < 0.0001) and therapeutic efficacy (RR = 1.37,95%CI [1.13,1.65], Z = 3.27, P = 0.001), and equal in safety (RR = 1.32,95%CI [0.47,3.7], Z = 0.53, P = 0.59). Gabapentin was similar to other neuromodulators in therapeutic efficacy (RR = 1.07,95%CI [0.87,1.32], Z = 0.64, P = 0.52), but its safety was better.

**Conclusion:**

Gabapentin is effective in the treatment of chronic refractory cough in both subjective and objective evaluations, and its safety is better than other neuromodulators.

## 1 Introduction

Chronic cough is defined as a cough that lasts for 8 weeks in adults and 4 weeks in children in the ERS guidelines [1,2], but with a more complicated situation in clinical work. The morbidity of chronic cough in adults is about 7%–11%, with an average of 10% [3,4]. This disease is more prevalent in Europe, America and Oceania, compared to Asia and Africa. The patients with chronic cough are mainly females, and the most common age for presentation was in the sixth decade [5]. Up to 40% of these patients can be refractory.

Chronic refractory cough is defined in the ERS guideline as a type of chronic cough which is persistent despite any investigation and treatment according to published practice guidelines [1]. Chronic refractory cough is not a serious or fatal disease, but more of a symptom of various diseases, so it hadn't attracted enough attention in the past. However, it indeed has a significant impact on patients' daily life, mental health and social communication, and even causes incontinence, cough syncope when it is serious. Nowadays, with the improvement of patients' requirements for quality of life, the treatment of chronic refractory cough has become a challenge in clinical practice, especially when the classic treatment is not ideal.

In the past decade, many researchers have indicated that chronic refractory cough, especially cough without obvious respiratory disease, may be induced more than just respiratory or throat diseases. They have noticed that chronic cough was very similar to chronic pain, and it, like chronic pain, may also be a neurological disease in some aspects [6,7]. Therefore, some drugs for neurological diseases, especially for chronic pain, have the potential of treating chronic cough [1,[6], [7], [8]]. Gabapentin, one of the drugs for neurological diseases, is a typical drug for chronic pain and epilepsy, and was first investigated in the treatment of chronic cough from 2005 [9]. Because of its underlying therapeutic mechanism for central sensitivity and less adverse effect, it has attracted more attention in the treatment of chronic refractory cough in recent years. However, the clinical trials of gabapentin for chronic refractory cough had some defects: fewer participants, lower research quality and greater bias, compared with other trials of gabapentin for chronic pain and epilepsy. The aim of this paper is to provide a theoretical basis for clinical treatment and further research through a systematic and comprehensive analysis of reported trials on gabapentin for chronic refractory cough.

## 2 Method

### 2.1 Search strategy

We searched PubMed, Embase (using Ovid platform), Cochrane Library, CNKI, VIP, Wangfang database and SinoMed from inception to September 28th, 2022. The keywords of searches included Gabapentin or 1-(Aminomethyl)cyclohexaneacetic Acid or Neurontin or Gabapentin Hexal Convalis or Gabapentin-Ratiopharm or Gabapentin Ratiopharm or Novo-Gabapentin or Novo Gabapentin or NovoGabapentin or PMS-Gabapentin or Apo-Gabapentin or Apo Gabapentin or ApoGabapentin or Gabapentin Stada, cough or coughs, chronic cough and chronic refractory cough in English platform, and 加巴喷丁, 1-（氨甲基）环己基乙酸, 咳嗽, 慢性咳嗽, 慢性难治性咳嗽 in Chinese platform. The retrieval process included comprehensive retrieval of literatures, deleting of duplicated literature, rough screening by reading titles and abstracts, screening by reading full texts, discussion of disputed literatures, and finally inclusion of eligible literature.

### 2.2 Selection criteria

Inclusion criteria was listed as follows: 1. Prospective study; 2. The language of publication was Chinese or English; 3. The research subjects were adults and met the diagnostic criteria of chronic refractory cough or unexplained chronic cough (UCC); 4. The intervention included gabapentin (if the intervention included other treatments, the control group also needed corresponding treatments), and the control measures could be placebo or other neuromodulators; 5. The outcomes should include one or more of the following: cough-specific quality-of-life score (LCQ score), cough severity score (VAS score), cough frequency, drug efficacy and adverse effect. 6. Relevant data can be extracted or transformed.

Exclusion Criteria were as follows: 1. Abstract in Chinese or English, text in other languages; 2. The subjects included pregnant or lactating women; 3. Gabapentin was used in both groups; 4. Data cannot be extracted even after transforming.

### 2.3 Data extraction

We did the work of literature screening, data extraction and quality evaluation by two researchers (Sheng Xie and Meiling Xie) independently, with the inclusion and exclusion criteria described above. In this process, any disputes should be resolved by discussion, otherwise should be referred to a third researcher (Yongchun Shen). Data of the type of study, number of participants, demographic baseline, course of disease, intervention, treatment, follow-up time and outcomes were included and analyzed.

### 2.4 Quality assessment

Tool of RoB 2(Version 6.2) was adapted for the assessment of literature quality and risk of bias. The tool included 7 items: random sequence generation, allocation concealment, blinding of participants and personnel, blinding of outcome assessment, incomplete outcome data, Selective reporting, other biases (e.g., residual effects in crossover trials, recruitment bias in cluster randomized trial, etc.). According to the specific situation, the literature was evaluated as low risk, high risk and uncertain risk in each item.

### 2.5 Data analysis

Review Manager 5.4.1 software was adopted for data analysis. The statistical strategies were as follows: continuous data were represented by Weighted Mean Difference (WMD) and 95% Confidence Interval (95%CI), while dichotomous data were represented by Relative Risk (RR) and 95%CI. Descriptive analysis was introduced while studies cannot be statistical analyzed. When heterogeneity test suggested that heterogeneity presented between studies (P ≤ 0.05, I^2^ ≥ 50%), random effect model was adopted for pooled analysis, and sensitivity analysis was performed to locate the source of heterogeneity. When heterogeneity test indicated that homogeneity presented between groups (P > 0.05, I2 < 50%), fixed effect model was adopted for pooled analysis. Funnel plot analysis was performed for publication bias if necessary.

## 3 Result

### 3.1 Literature screening

**Fig. 1:**
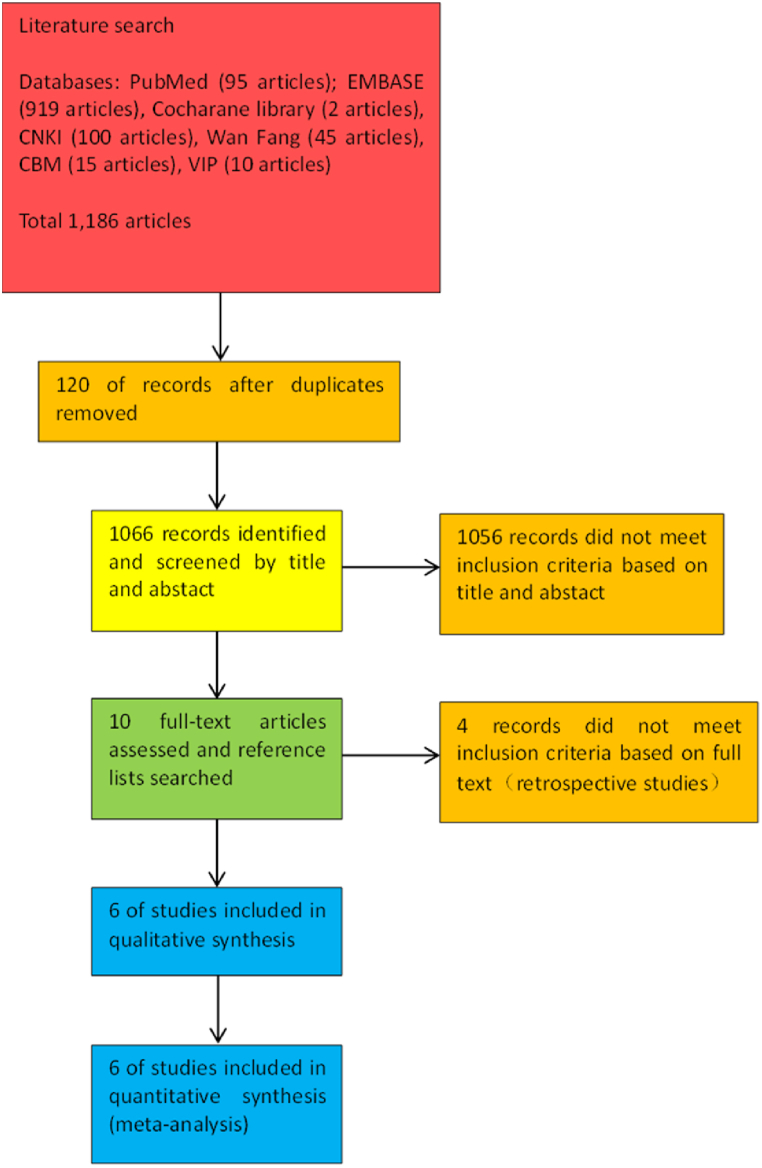
Literature screen process and outcomes.

Through the comprehensive retrieval of the databases mentioned above, a total of 1186 literatures were identified, and 6 full manuscripts were included after screening [[10], [11], [12], [13], [14], [15]]. The retrieval screening process is shown in Fig. 1.

### 3.2 Characteristics of included citations

All the six articles included were prospective studies, published from 2012 to 2019. A total of 536 adult participants were included, including the maximum subjects of 217 and the minimum subjects of 27. Of all the researches, five used gabapentin alone (maximum dose 1800mg/d, minimum dose 900mg/d) in the intervention group, and one used gabapentin combined with PPI (900mg/d +40mg/d). The duration of treatment and follow-up ranged from 4 weeks to 6 months. The basic characteristics of the six studies are shown in Table 1.

**Table 1:** Characteristics of included studies; *Y refer to Years; ** Mo refer to Months.

Citation	Study design	Number of subjects	Age		Course		Intervention measure		Duration of treatment	Duration of follow-up	Outcome indicators
			Intervention	Control	Intervention	Control	Intervention group	Control group			
Andrew Jay Bowen.et al., 2018	Prospective	28	N/A		7.93 Y*		Gabapentin（600 mg median; 300 mg mode, 1800 mg maximal）	Tricyclic antidepressant（30 mg median/mode, 50–60 mg maximal）	2 Mo+6 Mo	6 Mo	change of LCQ score, percentage improvement
Nicole M Ryan.et al., 2012	Prospective RCT (placebo compared)	52	62.7	60.9	3 Y	4 Y	Gabapentin（300 mg origin， 1800 mg maximal）	placebo	8 weeks	16 weeks	change of LCQ score in 8 weeks, change of cough frequency, LCQ score, VAS score,urge-to-cough score, LDQ score, theraputic efficacy
Ran Dong et al., 2019	Prospective open-labelled RCT (positive compared)	217	47.5	45.2	6.5 Mo**	7.5 Mo	Gabapentin(100 mg tid origin, maximal 300 mg tid)	Baclofen(10 mg tid origin, 20 mg tid maximal)	8 weeks	12 weeks	theraputic efficacy, change of cough symptom score, cough sensitivity, gastroesophageal reflux disease questionnaire score
Yiming Yu et al., 2019	Prospective	27	45	45	12 Mo		Gabapentin + PPI(100–300 mg tid+20 mg bid)	Baclofen + PPI(10–20 mg tid+20 mg bid)	4 weeks	4 weeks	theraputic efficacy
Heyao Kang et al., 2019	Prospective	156	N/A		N/A		Gabapentin(1500 mg maximal)	basic therapy	12 weeks	12 weeks	LCQ score, VAS score, cough frequency, theraputic efficacy
Cuibing Li et al., 2015	Prospective	56	50.5	52.2	32 Mo	39 Mo	Gabapentin(1800 mg maximal)	placebo	12 weeks	12 weeks	LCQ score, VAS score, cough frequency

### 3.3 Quality assessment and bias risk assessment

**Fig. 2:**
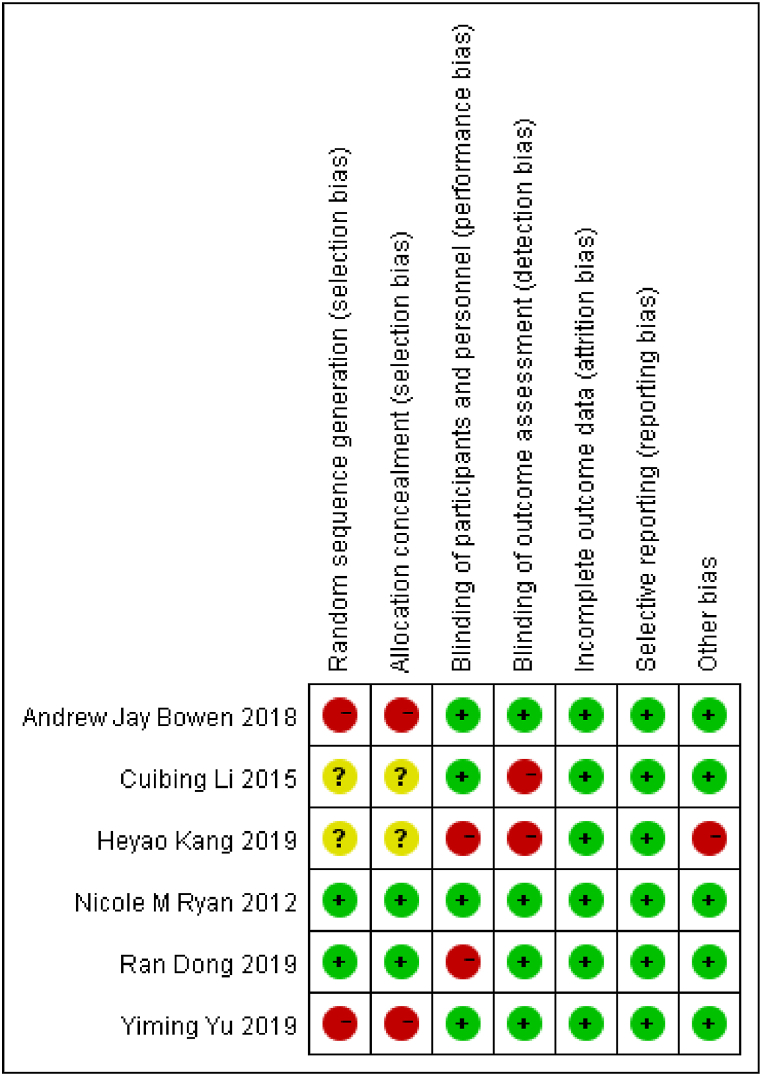
Quality assessment of included studies.

**Fig. 3:**
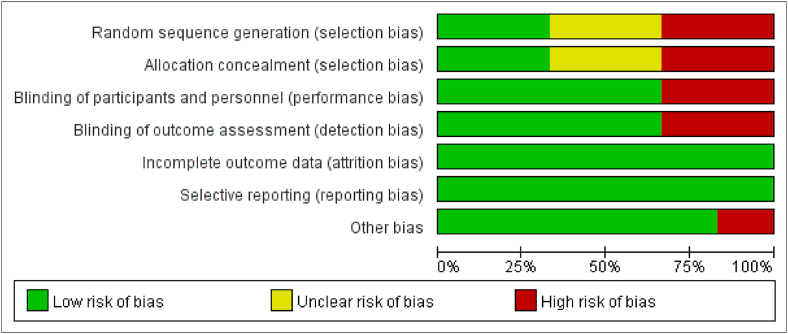
Bias risk assessment of included studies.

Among the six included studies, two studies were explicitly informed of the randomization methods [11,12], two studies were not randomized or allocation concealed [10,13], two studies were not informed of the method of randomization and allocation concealment [14,15]; two studies were not blinded in the experimental process [12,14], two studies were not blinded in the outcome measurement [14,15]; in one study [14], excluded and lost follow-up population was not reported or explained; in one study [15], outcome indicators were not clear. Quality assessment and bias risk assessment are shown in Fig. 2, Fig. 3.

### 3.4 Outcomes of meta-analysis

#### 3.4.1 Cough-specific quality-of-life score (LCQ score)

**Fig. 4:**
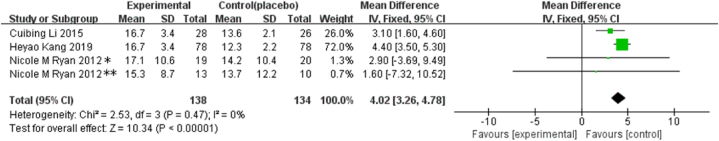
The LCQ score after using gabapentin and placebo. * refer to participants with CS, **refer to participants with No CS.

We chose LCQ score as the cough-specific quality-of-life score and three studies were included [11,14,15]. In the study of Nicole M Ryan et al. [11], extractable data were divided into Central sensitivities (CS) and non-central sensitivities (No CS), so we adopted both in the meta-analysis separately. The heterogeneity test showed no statistical significance (P = 0.47, I^2^ = 0%), so the fixed-effect model was adopted. As shown in Fig. 4, the LCQ score difference between the two groups was statistically significant (Z = 10.34, P < 0.00001), indicating that gabapentin could significantly improve the cough-specific quality of life in the patients with chronic refractory cough compared with placebo.

#### 3.4.2 Cough severity (VAS score)

**Fig. 5:**
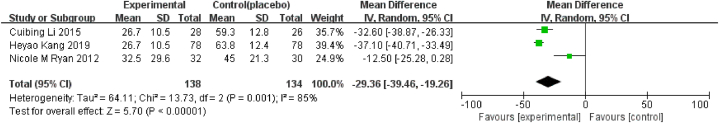
The VAS score after using gabapentin and placebo.

We chose VAS score as cough severity score. A total of 3 studies were included [11,14,15], and the random effect model was adopted (P = 0.001, I^2^ = 85%). As shown in Fig. 5, the VAS scores of the two groups showed a statistically significant difference (Z = 5.7, P < 0.00001), indicating that gabapentin could significantly improve the subjective cough severity of patients with chronic refractory cough compared with placebo.

#### 3.4.3 Cough frequency

**Fig. 6:**
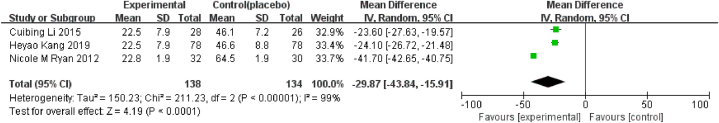
The cough frequency after using gabapentin and placebo.

Three studies were included in cough frequency [11,14,15], and the random effect model was adopted (P < 0.00001, I^2^ = 99%). As shown in Fig. 6, there was a statistically significant difference (Z = 4.19, P < 0.0001) in cough frequency between the two groups, indicating that gabapentin could significantly reduce the objective cough frequency of patients with chronic refractory cough compared with placebo.

#### 3.4.4 Therapy efficacy

Based on placebo or other neuromodulators as the control group, we divided the eligible studies into two groups for meta-analysis.

**Fig. 7:**
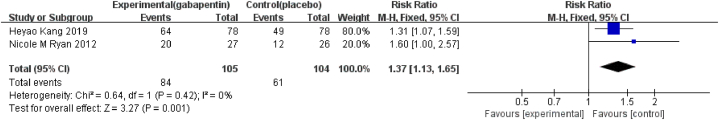
The therapy efficacy of gabapentin and placebo to chronic refractory cough.

There were two studies in which placebo was selected as control [11,14], and the fixed-effect model was adopted (P = 0.42, I^2^ = 0%). As shown in Fig. 7, there was a statistically significant difference (Z = 3.27, P = 0.001) in therapy efficacy between the two groups, indicating that gabapentin was more effective than placebo in the treatment of chronic refractory cough.

**Fig. 8:**
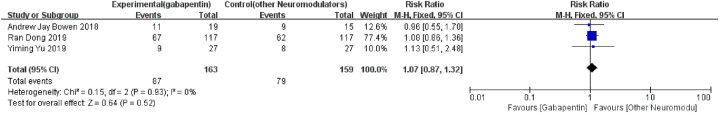
The therapy efficacy of gabapentin and other neuromodulators to chronic refractory cough.

There were three studies in which other neuromodulators were selected as control [10,12,13], and the fixed-effect model was adopted (P = 0.93, I^2^ = 0%). As shown in Fig. 8, there was no statistically significant difference (Z = 0.64, P = 0.52) in therapy efficacy between the two groups, indicating that gabapentin and other neuromodulators have similar efficacy in the treatment of chronic refractory cough.

#### 3.4.5 Therapy safety

Among the 6 included literatures, 5 literatures reported adverse effects [[10], [11], [12],^14^,^15^], including somnolence, dizziness, fatigue, gastrointestinal reactions (nausea, stomachache, vomiting, diarrhea, etc.), dry mouth, disorientation, confusion, blurred vision, memory loss, headache, depression, neuroticism, etc.

**Fig. 9:**
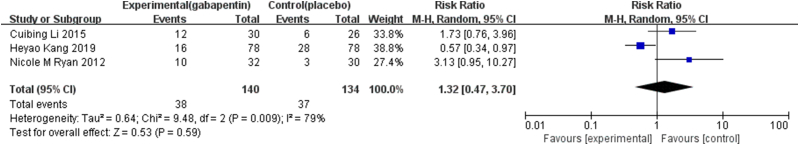
The side effect of gabapentin and placebo.

Three of the five studies selected placebo as control [11,14,15]. The details see Table 2. Meta-analysis was conducted and random effect model was adopted (P = 0.009, I^2^ = 79%). As shown in Fig. 9, there was no statistical significance (Z = 0.53, P = 0.59) in adverse reactions between the two groups, indicating that gabapentin did not increase the incidence of adverse effects compared with placebo.

**Table 2:** The side effect of gabapentin and placebo. The outcome is shown as experimental group: control group.

Citation	Dizziness	Fatigue	Gastrointestinal reactions	Dry mouth	Disorientation, confusion	Blurred vision	Memory decline	Headache	Depression	Neuroticism	Somnolence	Totol	P value
Nicole M Ryan.et al., 2012	3:1	3:1	4:2	2:1	2:0	1:0	1:0	1:0	0:1			10:3	0.059
Cuibing Li et al., 2015	1:0	1:1	2:2	2:1	2:0	1:0	1:0	1:1	1:1			12:6	≥0.05
Heyao Kang et al., 2019			9:13							5:9	7:11	16:28	–

**Fig. 10:**
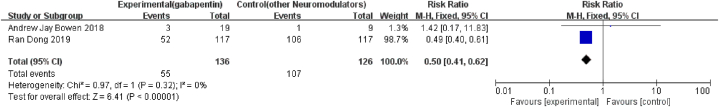
The side effect of gabapentin and other neuromodulators.

Two studies reported the comparison of adverse effects between gabapentin and other neuromodulators [10,12]. The details see Table 3. Meta-analysis was conducted and fixed-effect model was adopted (P = 0.32, I^2^ = 0%). As shown in Fig. 10, adverse effects in the two groups were statistically significant (Z = 6.41, P < 0.00001), indicating that gabapentin had a lower incidence of adverse effects compared with other neuromodulators.

**Table 3:** The side effect of gabapentin and other neuromodulators. The outcome is shown as experimental group: control group (P value).

Citation	Dizziness	Fatigue	Nausea	Diarrhea	Somnolence	Totol
Ran Dong et al., 2019	13:28 (0.010)	11:20 (0.083)	2:4 (0.408)	2:3 (0.651)	24:41 (0.013)	
Andrew Jay Bowen.et al., 2018						3:1

#### 3.4.6 Heterogeneity analysis

In the analysis of cough severity (VAS score), there was significant heterogeneity (P = 0.001, I^2^ = 85%) among the included studies. Sensitivity analysis showed that the heterogeneity decreased significantly (P = 0.022, I^2^ = 33%) when the studies of Nicole M Ryan et al. were excluded. We presumed that the sources of heterogeneity may include: 1. Literature quality; 2. The race of participants is different. However, no matter which study was removed, the outcome remained unchanged, suggesting that the results were relatively robust.

In the analysis of cough frequency, there was obvious heterogeneity (P < 0.00001, I^2^ = 99%) among the included studies. Sensitivity analysis was performed and studies were removed one by one. The heterogeneity decreased significantly (P = 0.84, I^2^ = 0%) when the studies of Nicole M Ryan et al. were excluded. We presumed that the sources of heterogeneity may include: 1. Literature quality; 2. The race of participants is different. However, no matter which study was removed, the outcome remained unchanged, suggesting that the results were relatively robust.

#### 3.4.7 Publication bias

Due to the small number of studies included in each research indicator, the validation of publication bias was not carried out. We presumed that publication bias may exist.

## 4 Discussion

Based on the above analysis, we can presume that gabapentin was superior to placebo in the therapy efficacy, improvement of cough-specific quality-of-life, reduction of cough severity and reduction of cough frequency in the treatment of chronic refractory cough, and the adverse effects were comparable to placebo. And compared with other neuromodulators, gabapentin was equal in the success rate of chronic refractory cough, but had a lower incidence of adverse effects.

Gabapentin was a typical medicine for chronic pain and epilepsy. Why is it effective in treating chronic refractory cough? It is related to the nerve function of cough. We will discuss this in the aspects of the nerve conduction pathway of cough, cough regulation mechanism and the role of gabapentin below.

### 4.1 The conduction pathway and regulation of normal cough reflex

**Fig. 11:**
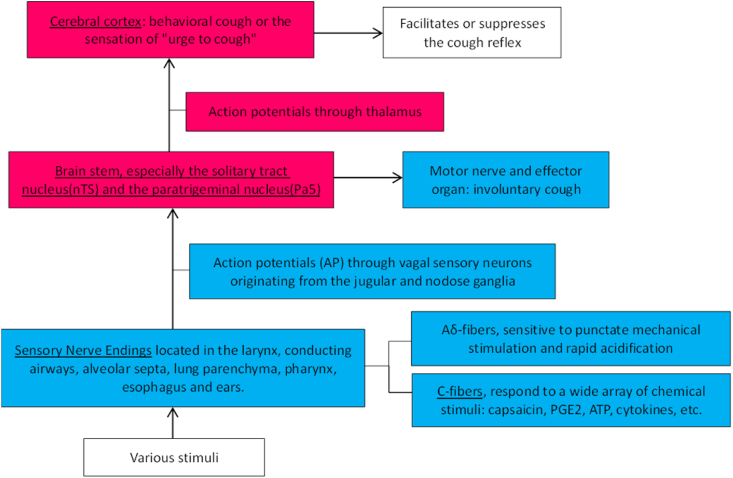
Conduction pathway of cough reflex [7,8,[16], [17], [18], [19]]. The underline part could be affected by gabapentin. The red parts refer to central nervous system, and the blue parts refer to peripheral nervous system. (For interpretation of the references to colour in this figure legend, the reader is referred to the Web version of this article.)

Cough is a protective mechanism of the airways. The conduction pathway of cough reflex see Fig. 11.

**Fig. 12:**
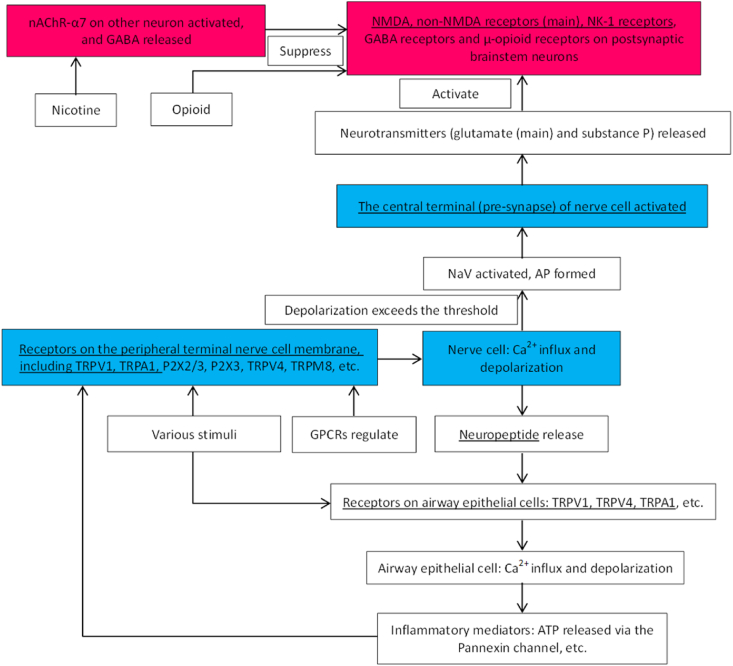
The receptors and regulation of cough reflex [[20], [21], [22], [23], [24]]. The underline part could be affected by gabapentin. The red parts refer to central nervous system, and the blue parts refer to peripheral nervous system. (For interpretation of the references to colour in this figure legend, the reader is referred to the Web version of this article.)

The receptors and regulation of cough reflex see Fig. 12.

### 4.2 Cough hypersensitivity syndrome, CHS

Although multiple factors can lead to chronic refractory cough, cough hypersensitivity syndrome is one of the most important mechanisms.

If (all or part of) the receptors on cough reflex are sensitized and respond to weak stimuli that do not lead to cough in normal condition, or overrespond to stimuli above the threshold, it indicates that the body is in a state of increased cough sensitivity, which is called cough hypersensitivity syndrome [25]. This sensitive state may be activated when patients get (respiratory) disease and lasts after disease cured. In the state of CHS, factors that can cause cough include talking, cold air and other stimuli which usually do not cause cough [20]. The patients of CHS usually describe a sensation of itchiness, irritation, and unpleasantness in the throat region or even describe it as “something physically stuck in the throat” [10,11,26], thus similar to laryngeal hypersensitivity syndrome [6].

So far, the exact mechanism of CHS hasn't be fully understood. Neuroinflammation maybe one of the main mechanisms of CHS, and is related to functional changes of TRPV1, TRPA1 and P2X3 [6]. The pathophysiological basis may be related to acid or non-acid fluid and gas reflux [20]. Existing studies have indicated that CHS has two aspects: peripheral sensitization and central sensitization. Peripheral sensitization refers to the increased excitability of peripheral sensory nerves to chemical and other stimuli. Peripheral sensitization may be related to the increase of endogenous inflammatory factors and the depolarization of downstream ion channels which are excited by G protein-coupled receptor, as well as the up-regulation of receptors [7]. The upregulated receptor is mainly TRPV1 [27], which is five times higher in patients with chronic cough than in normal people [28]. TRPV1 is a chemical gated calcium ion channel that is sensitive to capsaicin, and other factors that can stimulate it include pH change, temperature change and so on [29,30]. TRPV1 mainly exists in C fiber [7], and is also one of the downstream ion channels of G protein-coupled receptor. Central sensitization refers to the overrespond of the cough neural network of the central nervous system to peripheral stimuli. The mechanism of central sensitization in chronic cough is not very clear, but the process is similar to that in chronic pain. It is related to substance P, central glial cells, sensitization of secondary neurons and changes in the central descending system. The vagus nerve can also release some neurokinins under long-term inflammatory stimulation, which may also be related to central sensitization [7]. At the receptor level, central γ -aminobutyric acid receptor (GABA) and N-methyl-d-aspartic acid receptor (NMDA) are involved in central sensitization [31].

### 4.3 Chronic (refractory) cough and chronic pain

As described above, chronic (refractory) cough, like chronic pain, is a hypersensitive disorder of the nervous system. The similarities between the two diseases support the usage of gabapentin in chronic (refractory) cough. In previous studies, the following similarities had been found.

#### 4.3.1 Epidemiology

The overall prevalence of chronic cough is 9.6% [3], similar to the prevalence of chronic pain in Australia (8.5%) and Europe (7–8%) [32,33]. Patients of both chronic pain and chronic refractory cough are mainly female [[34], [35], [36]].

#### 4.3.2 Mechanism

Central and peripheral sensitization exists in both chronic pain and chronic cough [6]. Similar to referred pain and allodynia, symptoms like allotussia and hypertussia can be found in a variety of cough phenotypes [20]. The mechanism of referred pain and allodynia is mainly central sensitization, while inflammation of the lung and esophagus can also lead to discomfort in the larynx through central sensitization: C fiber of the esophagus and lung is stimulated and releases transmitters and peptides in the brain stem to sensitize cough reflex originating in the larynx, resulting in discomfort in the larynx [19,37].

#### 4.3.3 Neuroreceptors

A series of neuroreceptors exist in both chronic cough and chronic pain, details see Table 4.

**Table 4:** The receptors in chronic pain and chronic cough [6,16,28,[38], [39], [40], [41], [42], [43], [44], [45], [46], [47], [48], [49]]. Chronic (refractory) cough also has similarities with other neurological diseases except chronic pain (see Table 5).

	TRPV1	NK1	P2X3	NADM/non-NADM	Others (e.g. NaV)
In chronic pain	Several highly selective TRPV1 antagonists have been investigated for chronic pain	NK1 receptor antagonists can block behavioral responses to noxious (animal)	1.As a research target for the treatment of chronic pain, arthritis and other diseases. 2.P2X3 has been proved to be related to chronic pain (animal).	The antagonist ketamine is a analgesia and has both acute and prolonged effects on chronic neuropathic pain syndromes and symptoms of allodynia and hyperalgesia.	The antagonist lidocaine is a analgesia and anethesia
In chronic (refractory) cough	One highly selective TRPV1 antagonists is being investigated for chronic refractory cough	NK1 receptor antagonists can block behavioral responses to capsaicin-induced cough (animal)	P2X3 antagonists have an antitussive action, which is absence in capsaicin-induced cough, indicating that the action is independent of suppressing capsaicin effect (animal)	NMDA receptor activation play a predominant role in cough, and the modulatory role of non-NMDA receptors had also been found.	The upregulated NaVs on vagus nerve (NaV 1.7–1.9) increase the sensitivity of cough reflex

**Table 5:** Similarities in Chronic cough and other neurologic diseases [[50], [51], [52]].

	Trigger	Autosomal dominant hereditary sensory neuropathy	Neuralgia model
In chronic (refractory) cough	Exist in laryngeal hypersensitivity	Chief symptoms are cough associated with upper airway hypersensitivity and gastroesophageal reflux	Exist in UCC
In neurologic diseases	Exist in trigeminal neuralgia, etc.	A familial axonal sensory neuropathy	Exist in neuralgia

## 5 Mechanism of gabapentin

Gabapentin (1- (aminomethyl) cyclohexylacetic acid), chemical formula C9H17NO2, molecular weight 171.237 g/mol, is a lipophilic structural analog of the neurotransmitter g-aminobutyric acid (GABA) [53], with central action and peripheral analgesic effect [54,55]. Gabapentin is absorbed orally by diffusion and by the carrier-mediated, l-aminoacid transport system. Bioavailability of gabapentin is about 60% for a 300 mg dose, 40% for a 600 mg dose, and 35% for a 1600 mg dose [56]. The decrease in bioavailability of gabapentin with increased dose may be due to saturation of transport system [56]. Its bioavailability is not affected by food ingestion, but can be decreased by antacids (by 20%) when they are taken simultaneously or up to 2h after gabapentin administration. Gabapentin does not bind to plasma proteins and can cross the blood-brain barrier. The concentration of gabapentin in CSF equals 20% of the plasma concentration, and the concentration in brain tissue is about 80% of corresponding plasma concentration. Gabapentin is not metabolized and is excreted unchanged in urine. The elimination half-life is about 5–9 h. Gabapentin has a high frequency of administration due to its slow absorption, non-linear relationship between plasma concentration and dosage, and short half-life [57]. There were no significant pharmacokinetic interactions reported. The plasma gabapentin concentration of patients over 65 years old was twice that of young people [58]. The abrupt discontinuation of high-dose gabapentin can cause withdrawal reactions (irritability, agitation, anxiety, palpitation, and diaphoresis, etc.) for 1–2 days [56]. Dose escalation is recommended for initiation and dose tapering is recommended for cessation.

The mechanism of gabapentin has not been fully understood. So far, its pharmacological effects are thought to be binding α2δ1 auxiliary subunit (a auxiliary subunit of voltage-gated calcium channel, CaV), resulting analgesic effect. Its binding inhibits the transmembrane transport of α1 pore forming units of CaV (mainly Cav2.2 N type, existing in both central and peripheral synapses [59]) in the presynaptic membrane, thereby reducing the influx of calcium ions and the resulting release of neurotransmitters [20]. Furthermore, gabapentin also inhibits the axoplasmic transport of α2δ1 auxiliary subunits [20]. Gabapentin can also modulate TRP channels, NMDA receptors, protein kinase C, and inflammatory cytokines. Gabapentin can reduce the levels of TNF-α and IL-6 in spinal cord of L5 ligated rats, and has a dose-dependent effect [60]. Gabapentin can also reduce the expression of TRPV1, substance P and calcitonin gene-related peptide in lung tissues of post-infection cough rats, and reduce their airway neurogenic inflammation [61]. Gabapentin can act on supra-spinal regions to stimulate noradrenaline mediated descending inhibition, which contributes to its anti-hypersensitivity action in neuropathic pain [62]. The functional region of gabapentin in the central nervous system is not so clear, but it has been studied in some literatures. An fMRI study showed that gabapentin acted on multiple cortical and subcortical regions in rats, including the thalamus, periaqueductal gray matter (PAG), tegmental area, ectorhinal cortex, subiculum and amygdaloid nucleus, and it also showed that gabapentin can change the neural activity of the brain involved in nociceptive processing [63]. Another fMRI study demonstrated the complex effects of gabapentin on brain activation, the most pronounced one of which was a reduction in stimulus-induced brain deactivation following central sensitization [64].

Considering the similarities between chronic cough and chronic pain and the similar cortical response between airway stimulation and pain [[65], [66], [67]], the mechanism of gabapentin's inhibition of cough and pain may be similar. In the central nervous system, gabapentin can change the neural activity of the cerebral cortex involved in cough by NMDA receptors and CaV receptors and activate the descending inhibitory system, leading to the decrease of the central sensitivity of cough reflex. In the study of Bowen AJ et al., gabapentin was more effective in the UCC patients with paroxysmal spasms, dysphonia or cough triggered by talking, and considering that throat irritation and talking-triggered cough were the symptoms indicated the high sensitivity of central nervous system, this outcome may reflect the central mechanism of gabapentin for chronic refractory cough [10,11]. Meanwhile, gabapentin could reduce peripheral sensitivity of cough reflex by modulating peripheral TRP channels and inflammatory factors in cough-related sites.

About safety, the concentration of gabapentin in brain tissue equals 80% of plasma, so its adverse effects mainly affect the central nervous system. Studies have shown that gabapentin is safe in most times. The most serious adverse effects reported were rhabdomyolysis and acute renal insufficiency in 2 diabetic patients, both of which recovered after treatment [68,69]. In a retrospective case series, 48 patients with overdosing gabapentin (vary from 600 mg to 3000 mg) suffered no or only mild adverse effects, and the highest tolerated dose was 48g [70]. Gabapentin is therefore safe in oral administration, but we should pay more attention when it is used in the elderly, in patients with renal insufficiency, and when used with antacids.

We should ensure that safe dose of gabapentin must be guaranteed, but on the other hand, sufficient dose may also be one important factor affecting its efficacy. In the study of Bowen AJ et al. [10], 59% of patients in the gabapentin group completed the 6-month treatment when the drug dosage was increased (the main reason for withdrawal was tachyphylaxis rather than side effects), and the average increase in LCQ score (5.4) was doubled compared to 2-month treatment (2.48). It was also higher than the average increase in LCQ score of the 2-month tricyclic antidepressant group (3.46). Only three (20%) of the tricyclic antidepressant group in this study completed 6-month treatment (also mainly because of tachyphylaxis), reflecting the tolerance and long-term efficacy of gabapentin was better than other neuromodulators, particularly tricyclic antidepressants.

There were some defects when gabapentin was used for chronic refractory cough. In the study of Ryan et al. changes in LCQ score and cough frequency returned to baseline after treatment withdrawal, and VAS score was even higher than the baseline, indicating that the efficacy of gabapentin was poorly sustained after withdrawal [11]. However, in the study of Dong Ran et al. the recurrent rate in gabapentin group was only 3% 2 weeks after complete cessation of gabapentin [12]. Therefore, the efficacy sustenance of gabapentin after withdrawal needs more research. Furthermore, the efficacy of gabapentin may decrease after a period of use, which may be the result of the downregulation of glutamate transporter 1 (necessary for initiating noradrenergic signaling) in the locus coeruleus [71]. Therefore, gabapentin was not so efficient in long-term treatment compared to initial use. In addition, SF-36 scores in the gabapentin group were also lower than those in the control group in the study of Ryan et al. suggesting that while cough-specific quality of life improved, patients' overall quality-of-life decreased. Ryan et al. did not explain this result, but we assumed that it may be related to the adverse effects of gabapentin.

There are several shortcomings in this study. There is very little eligible literature included in this study, and even less for each meta-analysis. Moreover, due to the small number of RCTs studying gabapentin in the treatment of chronic refractory cough, the included studies are not all RCTs, and the quality of some studies is not very high, so there are risks of publication bias and other bias. The basic disease status of patients in different studies were not the same or similar, which may increase the risk of bias. However, as the patient status varies and the results are robust by sensitivity analyze, the results may be applied to more patients.

There are some aspects we think need to be studied on the use of gabapentin for chronic refractory cough in future. The first one is the peripheral effect of gabapentin for chronic refractory cough. The study of Ryan NM et al. showed that gabapentin had a poor effect on capsaicin-induced cough, which was ascribed to its poor effect on the peripheral sensitivity [11]. However, in the study of Dong R et al., C2 and C5 were significantly increased in gabapentin group, which indicated that gabapentin could decrease the sensitivity of peripheral nervous system [12]. Although Dong R et al. ascribed the discrepancy to the difference of the recruited patients, the role of gabapentin in peripheral sensitivity of chronic refractory cough and whether gabapentin is more suitable for patients with peripheral sensitivity compared to those who without should be studied in subsequent trials. And the recurrence rate of cough in patients after drug withdrawal was also different as mentioned above, so the sustenance of gabapentin treatment efficacy can also be part of the future study. In Dong R et al. ‘s study, there was no statistically significant difference in the efficacy of gabapentin in cough patients with acid and non-acid reflux. Yu Y et al. found that gabapentin was more effective in patients with RSI>19, while patients with RSI>19 had more proximal reflux (closer to the throat), non-acid reflux and gas reflux [13]. These differences and mechanisms may also provide a direction for future research.

In conclusion, we presume that gabapentin can be used as the first choice in the treatment of chronic refractory cough for its better efficacy and less adverse effects. However, as there are still some discrepancies between studies and too little RCTs, further research is needed to support this conclusion.

## Author contribution statement

Sheng Xie: Conceived and designed the experiments; Performed the experiments; Analyzed and interpreted the data; Contributed reagents, materials, analysis tools or data; Wrote the paper.

Meiling Xie, Yongchun Shen: Performed the experiments; Analyzed and interpreted the data; Contributed reagents, materials, analysis tools or data; Wrote the paper.

Deyun Cheng: Conceived and designed the experiments; Analyzed and interpreted the data; Contributed reagents, materials, analysis tools or data; Wrote the paper.

## Data availability statement

Data will be made available on request.

## Declaration of interest’s statement

The authors declare no competing interests.

## Additional information

No additional information is available for this paper.
